# Cardiac dysfunction in pediatric patients on renal replacement therapy

**DOI:** 10.1007/s00467-012-2214-9

**Published:** 2012-06-12

**Authors:** Dorota Polak-Jonkisz, Malgorzata Sobieszczanska

**Affiliations:** 1 Department of Pediatric Nephrology, Wroclaw Medical University, Borowska 213, Wroclaw, 50-556 Poland; 2 Department of Pathophysiology, Wroclaw Medical University, Marcinkowskiego 1, Wroclaw, 50-372 Poland

Dear Editor,

We read with great interest the article by Malatesta-Muncher et al. entitled “Early cardiac dysfunction in pediatric patients on maintenance dialysis and post kidney transplant”, where the authors discussed the problem of “cardiorenal syndrome” which seems to be of high predictive importance in young patients with chronic kidney disease (CKD) on renal replacement therapy [1].

The authors investigated heart changes, using imaging techniques for structural study and sensitive biomarkers to assess myocardial function and metabolism, in order to examine the association of these parameters with left ventricular hypertrophy (LVH). They calculated that cardiac magnetic resonance (CMR) and MR spectroscopy (MRS) allow the detection of cardiac dysfunction earlier. They also concluded that young patients with advanced CKD and normal ejection fraction demonstrated early cardiac impairments accompanied by increased LV mass index, which suggested a development of maladaptive hypertrophy.

In this regard, we would like to pay attention to our observations made in children with CKD, which revealed the usefulness of a non-invasive 87-electrode electrocardiographic method, BSPM (body surface potential mapping), in early detection of intraventricular conduction disturbances of various degrees.

We have used isochrone maps that precisely reflect ventricular activation time (VAT maps). In the examined CKD children treated conservatively [2] or with renal replacement [3], the maps showed a varied abnormal distribution of prolonged VAT values, which suggested obstacles in the intraventricular conduction pathway. In patients hemodialyzed up to 12 months, the pattern specific for left bundle branch block (LBBB) was found, and after renal transplantation essential alleviation occurred, with a transformation to the left anterior fascicle block pattern. The patients hemodialyzed over 12 months manifested more significant disturbances of the complete LBBB type that were normalized after transplantation to the incomplete LBBB pattern. It is noteworthy that all patients demonstrated neither conduction abnormalities on standard ECG, nor left ventricular hypertrophy or compromised cardiac systolic function in echocardiography.

Our results, being in strong relevance to the findings of Malatesta-Muncher et al., suggest that children with CKD, regardless of mechanisms responsible for LVH, adaptive or maladaptive, are at risk of developing progressive abnormalities in the intraventricular heart conduction system. Such disturbances of electrical activation propagation lead to delayed ventricular depolarization, which results in hemodynamic dyssynchrony and, consequently, with simultaneous left ventricular hypertrophy, worsen the global systolic function of the heart. Bearing this in mind, any possibilities enabling detection of subclinical cardiac dysfunction in CKD seem beneficial.
